# S100B is required for maintaining an intermediate state with double-positive Sca-1+ progenitor and vascular smooth muscle cells during neointimal formation

**DOI:** 10.1186/s13287-019-1400-0

**Published:** 2019-09-23

**Authors:** Yan Wu, Xin Liu, Ling-Yun Guo, Lei Zhang, Fei Zheng, Shan Li, Xing-Yuan Li, Ye Yuan, Yu Liu, Yu-wen Yan, Shi-You Chen, Jia-Ning Wang, Jin-xuan Zhang, Jun-Ming Tang

**Affiliations:** 1 Department of Physiology, School of Basic Medicine Science, Hubei University of Medicine, Shiyan, 442000 Hubei China; 2 Institute of Clinical Medicine and Department of Cardiology, Renmin Hospital, Hubei University of Medicine, Shiyan, 442000 Hubei China; 3 Laboratory Animal Center, Hubei University of Medicine, Shiyan, 442000 Hubei China; 4 Institute of Biomedicine and Key Lab of Human Embryonic Stem Cell of Hubei Province, Hubei University of Medicine, Shiyan, 442000 Hubei China; 5 Department of Biochemistry, School of Basic Medicine Science, Hubei University of Medicine, Shiyan, 442000 Hubei China; 6 Department of Physiology & Pharmacology, The University of Georgia, Athens, GA 30602 USA

**Keywords:** S100B, Sca-1, Intermediate state, Neointima, SDF-1α, NF-κB

## Abstract

**Introduction:**

Accumulation of vascular smooth muscle cells (VSMCs) within the neointimal region is a hallmark of atherosclerosis and vessel injury. Evidence has shown that Sca-1-positive (Sca-1+) progenitor cells residing in the vascular adventitia play a crucial role in VSMC assemblages and intimal lesions. However, the underlying mechanisms, especially in the circumstances of vascular injury, remain unknown.

**Methods and results:**

The neointimal formation model in rats was established by carotid artery balloon injury using a 2F-Forgaty catheter. Most Sca-1+ cells first appeared at the adventitia of the vascular wall. S100B expressions were highest within the adventitia on the first day after vessel injury. Along with the sequentially increasing trend of S100B expression in the intima, media, and adventitia, respectively, the numbers of Sca-1+ cells were prominently increased at the media or neointima during the time course of neointimal formation. Furthermore, the Sca-1+ cells were markedly increased in the tunica media on the third day of vessel injury, SDF-1α expressions were obviously increased, and SDF-1α levels and Sca-1+ cells were almost synchronously increased within the neointima on the seventh day of vessel injury. These effects could effectually be reversed by knockdown of S100B by shRNA, RAGE inhibitor (SPF-ZM1), or CXCR4 blocker (AMD3100), indicating that migration of Sca-1+ cells from the adventitia into the neointima was associated with S100B/RAGE and SDF-1α/CXCR4. More importantly, the intermediate state of double-positive Sca-1+ and α-SMA cells was first found in the neointima of injured arteries, which could be substantially abrogated by using shRNA for S100B or blockade of CXCR4. S100B dose-dependently regulated SDF-1α expressions in VSMCs by activating PI3K/AKT and NF-κB, which were markedly abolished by PI3K/AKT inhibitor wortmannin and enhanced by p65 blocker PDTC. Furthermore, S100B was involved in human umbilical cord-derived Sca-1+ progenitor cells’ differentiation into VSMCs, especially in maintaining the intermediate state of double-positive Sca-1+ and α-SMA.

**Conclusions:**

S100B triggered neointimal formation in rat injured arteries by maintaining the intermediate state of double-positive Sca-1+ progenitor and VSMCs, which were associated with direct activation of RAGE by S100B and indirect induction of SDF-1α by activating PI3K/AKT and NF-κB.

**Electronic supplementary material:**

The online version of this article (10.1186/s13287-019-1400-0) contains supplementary material, which is available to authorized users.

## Introduction

Coronary artery disease (CAD) is mainly attributed to atherosclerosis and seriously threatens human health because of high morbidity and mortality [1]. Accumulation of vascular smooth muscle cells (VSMCs) within the neointimal region is a hallmark of atherosclerosis and vessel injury [2]. Published data have shown that adventitial (Adv) Sca-1-positive (Sca-1+) progenitor cells have multiple differentiation potential into VSMCs and likely contribute to intimal lesions in vivo [3]. Percutaneous coronary intervention (PCI) is one of the most effective treatment options of CAD and is widely used [4]. Despite this, it is still necessary to recognize VSMC assemblages and PCI postoperative restenosis to provide novel prophylactic and therapeutic drug targets for vascular diseases, especially CAD.

Sca-1+ progenitor cells reside in the inner side of the vascular adventitia; they originate neither from the bone marrow nor from the circulating cells based on genetic tracing and bone marrow transplantation [5–7]. Later studies reveal that Sca-1+ progenitor cells occur late during vascular development after the arterial media is fully formed, whereas VSMCs have already acquired a differentiated phenotype in the meantime [8, 9]. Differentiated VSMCs in the outer media can migrate into the inner adventitia and lose expression of VSMC markers, gain expression of progenitor cell markers, and contribute to a subpopulation of Sca-1+ progenitor cells [10, 11]. However, the underlying mechanisms, especially in the circumstances of vascular injury, remain unknown.

S100B, a member of the S100 multigenic family expressing small (9 kDa and 14 kDa) Ca^2+^-binding proteins of the EF-hand type, is mainly localized in astrocytes under normal physiological conditions [12]. Previous evidence has suggested that S100B was involved in neointimal formation mediated by VSMC phenotypic transformation through interaction with receptor for advanced glycation end-products (RAGE) [13]. Recent studies have shown that S100B induced migration and infiltration of inflammatory cells, leading to human diseases such as cerebral ischemic disease and muscular dystrophy [14, 15]. Of interest, S100B/RAGE have shown multiple roles in regulating stem cell function including proliferation, differentiation, and stemness under physiological and pathological conditions [16–19]. Thus, these results suggested a potential link between S100B and Sca-1+ cells in mediating neointimal formation.

Published data have shown that migration of stem cells into the intima plays a key role in neointimal formation [8–11]. Stromal cell-derived factor-1 α (SDF-lα), one of the crucial signal molecules and landmarks of cell migration, was actually involved in triggering migration of Sca-1+ progenitor cells into the neointima by activating its receptor CXCR4 in the process of neointimal formation after vessel damage [20]. Considering the traits of common action of S100B and SDF-lα as inflammatory factors, S100B levels reached the peak at 2–6 h after tissue injury [21, 22], while SDF-lα expressions peaked 24 h after heart and vessel injury [23, 24], indicating that S100B could have a potential role in inducing SDF-lα expression in the injured vessels, and involving in the development of neointimal formation through SDF-lα signaling.

In this study, we found that Sca-1+ progenitor cells first increased in the inner adventitia of the vascular bed followed by in the neointima; S100B expression increased in a time-dependent manner in the sequence of adventitia, media, and neointima during neointimal formation. Furthermore, S100B could play a crucial role in maintaining the intermediate state with double-positive Sca-1+ progenitor cells and VSMCs during injury-induced neointimal formation, which was associated with RAGE and SDF-1/CXCR4 signaling.

## Methods

### Animals

According to the Guide for the Care and Use of Laboratory Animals (Chinese version), all animals were raised in specific-pathogen-free (SPF) grade animal laboratories. The model preparations of balloon-injured carotid artery in rats were approved by the Committee of Experimental Animals Care of Hubei University of Medicine.

### Human samples

The study was performed in strict adherence with the ethical guidelines for biomedical research involving human subjects in China and was approved by the Institutional Review Board of Shiyan Renmin Hospital, Hubei University of Medicine. Written informed consent was obtained from all participating individuals. Human umbilical cords were collected before disposal after babies were born in Shiyan Renmin Hospital.

### Human umbilical cord mesenchymal stem cell isolation and culture

To isolate human umbilical cord mesenchymal stem cells (hUCMSCs), the media layer of fresh human umbilical cord was carefully removed; the adventitial tissues were collected and cut into 0.5-mm-thick pieces for culture on 10-cm plates in a CO_2_ incubator at 37 °C for 3 h before adding the stem cell growth medium [25]. After 5–7 days of incubation, the migrated cells from the adventitial tissues were digested with 0.25% pancreatic enzymes and collected for purification. The ratio of Sca-1^+^ progenitor cells in hUCMSCs was evaluated by immunofluorescence assay of Sca-1.

### VSMC culture

Primary VSMCs were cultured from the aorta of Sprague–Dawley rats (280–300 g) as previously described [13]. VSMCs were cultured and reached 60% confluence; the cells were transfected with Ad-Null, Ad-S100B, or Ad-sh100B for 24 h. The culture media were changed thereafter.

To detect the relationship between S100B and SDF-1α, the cells were transfected with Ad-S100B with different multiplication of infection (MOI) for 3 days.

For experiments on inhibition, VSMCs were transduced with Ad-Null, Ad-S100B, or Ad-sh100B followed by treatment with vehicle (CTL) or selective inhibitors of NF-κB (PDTC, 50 μM), PI3K (WM, wortmannin, 50 nM), MAP kinase (PD, PD98059, 50 μM), or p38 MAPK (SB, SB203580, 30 μM).

To observe the effects of S100B on Sca-1+ progenitor cell migration, conditioned medium (CM) was prepared from the VSMC cultures. Briefly, the corresponding CM of VSMCs was collected 3 days after transfecting with Ad-Null (CM-Ctrl) and Ad-S100B (CM-S100B) as previously described [13].

### Carotid artery balloon injury model and adenoviral gene transfer

Carotid artery balloon injury model was performed as described previously [26] with a 2F-Forgaty catheter (CA92614-5686, Edwards Life sciences LLC Co.). The arterial segment from the proximal edge of the omohyoid muscle to the carotid bifurcation was washed with saline and incubated with 100 μl of saline or adenovirus expressing S100B shRNA (5 × 10^9^ pfu) or AMD3100 (10 μM) via a fixed catheter for 20 min [27]. Seven or 14 days later, the rats were euthanized with isoflurane, and the balloon-injured and adenovirus-dwelled segments were perfused with saline and collected for follow-up detection and analysis.

### Histomorphometric analyses

Common carotid artery segments were cut with serial sectioning to a thickness of 5 μm. Hematoxylin and eosin (H&E) staining was carried out for morphometric analyses. The images of five fields of view cross sections were randomly captured using a 80i Nikon microscope (Nikon, Inc). Using special software (Image-Pro Plus, Media Cybernetics), areas of the lumen, internal elastic lamina, and external elastic lamina were determined by two double-blinded pathologists, and the ratio of intimal and medial area (I/M) was calculated with following formula: I/M ratio (%) = [IEL area-lumen area]/[EEL area-IEL area] × 100 (%) [13].

### Immunohistochemistry

After rehydration and antigen retrieval, artery sections in different time points were blocked with 5% goat serum and permeabilized with 0.01% Triton X-100 in PBS, and incubated with S100B (ab52642, Abcam) antibody at 4°C overnight followed by incubation with HRP-conjugated secondary antibody for immunochemistry staining. The sections were counterstained with hematoxylin. Using special software (Image-Pro Plus, Media Cybernetics), the gray value of the interest protein expression in the target area was extracted precisely, and mean gray value (MGV) is calculated. Ratio of the interest protein expression in target area analyzed with the following formula: the ratio = MGV in treatment group/MGV in control group × 100 (%).

### Immunofluorescent staining

To confirm the dynamic process of Sca-1+ progenitor cell migration from the adventitia to intima during the process of neointimal formation, the artery sections from different time points were incubated with Sca-1 (ab4336, Abcam) antibody followed by fluorescent dye-conjugated secondary antibody (Jackson ImmunoResearch) and counterstained with DAPI (Sigma) [28].

To detect SDF-1α expression in the VSMCs of injured vessels using co-immunofluorescent staining, the artery sections from different time points were incubated with α-SMA (sc-130616, Santa Cruz, 1:100) and SDF-1α (ab9797, Abcam, 1:100) antibodies followed by fluorescent dye-conjugated secondary antibody and counterstained with DAPI.

To observe the intermediate state of double-positive Sca-1+ progenitor and VSMCs using co-immunofluorescent staining of Sca-1 and α-SMA, the artery sections were incubated 7 or 14 days after treatment with Ad-shS100bB or AMD 3100; subsequently, corresponding fluorescent dye-conjugated secondary antibody was used, and the tissues were counterstained with DAPI.

To observe the expression of RAGE and CXCR4 in Sca-1+ progenitor cells 7 days after the vessels were injured in vivo, the sections were rinsed with PBS and fixed with 4% paraformaldehyde, then blocked with 5% goat serum, permeabilized with 0.01% Triton X-100 in PBS, and incubated with anti-Sca-1 (ab4336, Abcam), anti-RAGE (ab54741, Abcam), and anti-CXCR4 (ab124824, Abcam) followed by TRITC-conjugated secondary antibody (Jackson ImmunoResearch) and counterstained with DAPI.

To observe the expression of RAGE and CXCR4 in Sca-1+ progenitor cells in vitro, Sca-1+ stem cells cultured on coverslips were rinsed with PBS and fixed with 4% paraformaldehyde, then blocked with 5% goat serum, permeabilized with 0.01% Triton X-100 in PBS, and incubated with anti-Sca-1, anti-RAGE, and anti-CXCR4 followed by TRITC-conjugated secondary antibody and counterstained with DAPI.

The images of five fields of view cross sections were randomly captured using a 80i Nikon fluorescence microscope (Nikon, Inc). Using Image-Pro Plus software (Media Cybernetics), the fluorescence intensity value of the interest gene expression within the target area is precisely extracted, and mean fluorescence intensity value (MFI) is calculated. Ratio of the interest protein expression in target area was analyzed with the following formula: the ratio = MFI in treatment group/MFI in control group × 100 (%).

### Nuclear translocation analysis

To confirm if the effects of Ad-S100B on SDF-1α expression involved the activation of NF-kBp65 and p52, the nuclear translocation of NF-kBp65 and p52 was analyzed in VSMCs exposed to Ad-S100B. The cells cultured on coverslips were rinsed with PBS and fixed with 4% paraformaldehyde, then blocked with 5% goat serum, permeabilized with 0.01% Triton X-100 in PBS, and incubated with anti-NF-kBp65 (sc-109, 1:250, Santa Cruz) and anti-NF-kBp52 (sc-7386, 1:250, Santa Cruz) antibodies, followed by TRITC or FITC-conjugated secondary antibody (Jackson ImmunoResearch) and counterstained with DAPI.

### Western blotting

Cells samples were then mingled with RIPA buffer containing protease inhibitor mix for extracting total proteins. Protein concentration was measured using the BCA Protein Assay Kit. Briefly, 20-μg protein samples were separated on 4–12% SDS-polyacrylamide gels and electro-transferred onto PVDF membranes (Bio-Rad). The membranes were incubated at 4 °C overnight with antibodies against S100B (ab52642,1: 500, Abcam); SDF-1α (ab9797, 1:500, Abcam) or AKT (#9272 s, 1:500, Cell Signaling); pAKT (#4058 s, 1:500, Cell Signaling); extracellular signal-regulated kinase (ERK1/2, SC-94, SC-154, 1:500, Cell Signaling); pERK1/2 (4370 s, 1:500, Cell Signaling); mitogen-activated protein kinase (p38MAPK, #9211, 1:500, Cell Signaling); p-p38MAPK (SC-7973, 1:500, Cell Signaling); or α-Tubulin (T6074, Sigma) in blocking buffer containing 5% milk followed by incubation with HRP-conjugated secondary antibody (Sigma).

### Transwell invasion assays

To determine the functions of S100B and SDF-1α in regulating Sca-1+ progenitor stem cell migration, CM-Ctrl or CM-Ad-S100B were used to explore the cells’ migration using the transwell system with or without neutralizing antibodies, namely S100B, RAGE inhibitor SPF-ZM1, or AMD3100. After 48-h incubation, cells were fixed with 100% methanol and stained with crystal violet (0.1%). The cells within the upper membranes were removed, and those within the bottom membranes were imaged under a light microscope and mounted for quantitative analysis. Images of three different fields of view of each of the transwell membrane were acquired with an optical microscope under × 10 magnifications. Each of the three independent experiments was repeated in triplicate. Migration indexes were calculated as follows: treatment group cells number/control group cells number.

### Statistical analysis

All data were expressed as mean ± SD and then evaluated with two-tailed, unpaired Student’s *t* test or compared by one-way ANOVA followed by the *t* test. *P* < 0.05 was considered to indicate statistical significance.

## Results

### Sca-1+ progenitor cell migration and S100B expression during balloon injury-induced neointimal formation

**Fig. 1:**
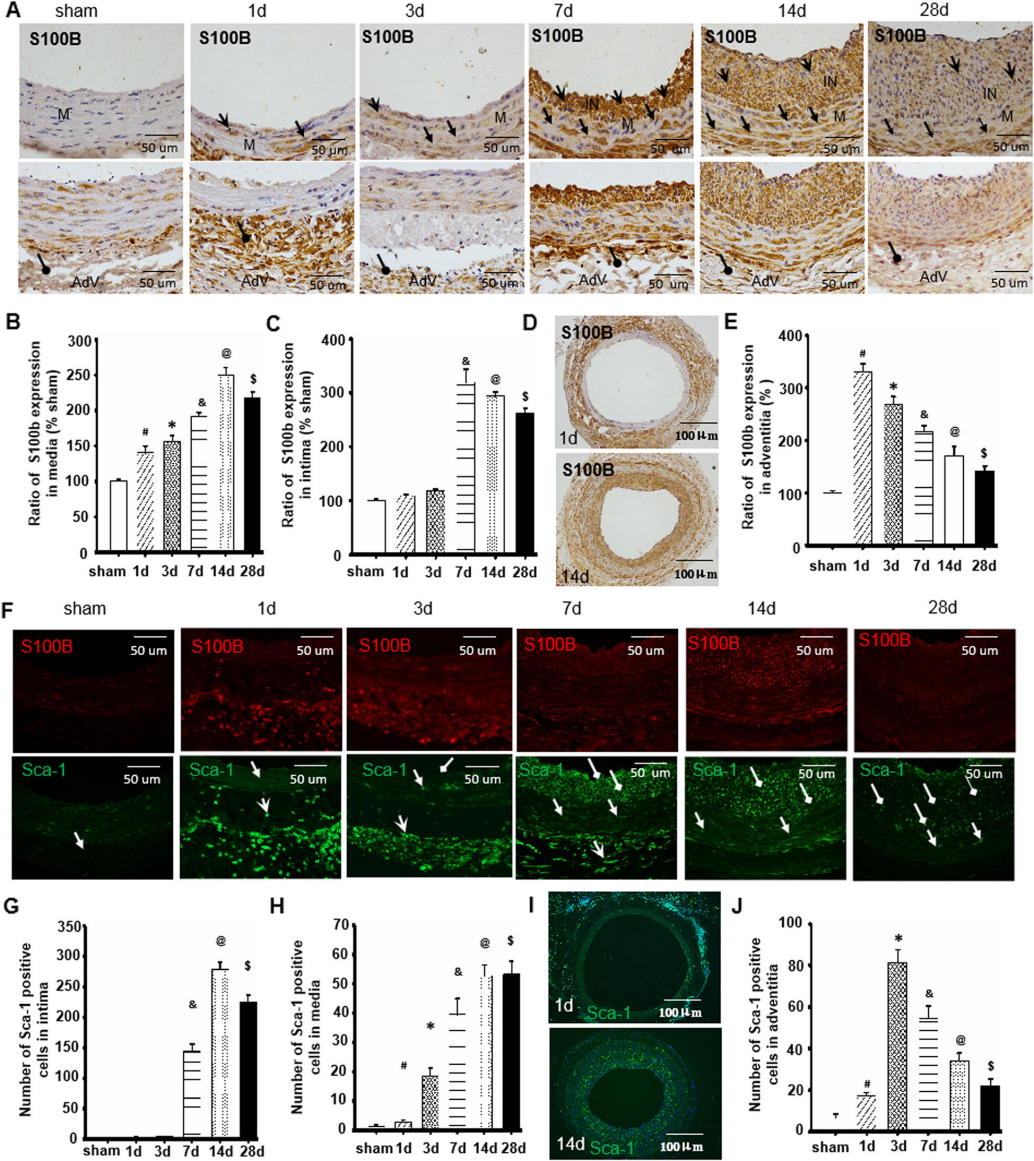
Sca-1-positive progenitor cell migration and S100B expression during balloon injury-induced neointimal formation. The experiment preliminarily demonstrated the traits of distribution of both Sca-1+ progenitor cells and S100B expression in the vessel wall during the process of neointimal formation after vascular injury and identified the correlation between them. **a** Rats underwent carotid artery balloon injury. Representative sections of sham-operation and injured arteries at the indicated time points were stained with S100B. Open arrow indicates the neointima area, forked tail arrow indicates the media, and round arrow indicates the adventitia. **b**, **c** Semi-quantitative analysis of optical density value of immunohistochemical staining of S100B was determined within the media or intima of sham-operated and injured arteries by image-Pro Plus software. The percentage of S100B expression at the media or intima at the indicated time was calculated and compared to the sham group. **d** Representative whole image of immunohistochemical staining of S100B on the first and 14th day after injury. **e** Percentage of S100B levels at the vessel wall adventitia were determined at the indicated time and compared to the sham group by image-Pro Plus software. **f** Typical image of immunofluorescence staining with Sca-1 in sham-operated and injured arteries at the indicated time. Red fluorescence indicates Sca-1; blue fluorescence indicates DAPI-labeled nucleus. Round arrow indicates the neointima area, open arrow indicates the media, and forked tail arrow indicates the adventitia. **g**, **h** The number of Sca-1+ progenitor cells at the media or intima at the indicated times was calculated and compared to the sham group. **i** Representative whole image of immunofluorescence staining of Sca-1 cells on the first and 14th day after injury. Red fluorescence indicates Sca-1; blue fluorescence indicates DAPI-labeled nucleus. **j** The number of Sca-1+ progenitor cells at the vessel wall adventitia were determined at the indicated time and compared to the sham group by image-Pro Plus software. *n* = 6, ^#^*P* < 0.05 compared to sham group; *P < 0.05 vs. first day after injury; ^&^*P* < 0.05 vs. second day after injury; ^@^*P* < 0.05 vs. seventh day after injury; ^$^*P* < 0.05 vs. 14th day after injury

To explore the relationship between Sca-1+ progenitor cell migration and S100B expression during injury-induced neointimal formation, we detected the expressions of Sca-1 and S100B in balloon-injured carotid artery by immunohistochemical staining. As shown in Fig. 1a–c, accompanied by the progressive increase of neointimal formation in the injured artery, S100B expressions were gradually induced in a time-dependent manner and showed increased expression in the sequence of adventitia, media, and neointima. Indeed, compared with the sham group, the increased S100B expressions were first found in the artery on day 1 after the injury. Within the injured artery, the S100B expressions were greater at the adventitia of the vessel wall than at the media on the first day after injury (Fig. 1d, e). By contrast, S100B levels at the adventitia were lower than at the media or neointima 14 days after injury (Fig. 1d, e).

Importantly, following the changed characteristics of S100B in the injured artery, the numbers of Sca-1+ progenitor cells gradually increased at the adventitia of the vessel wall in a time-dependent manner and peaked on the seventh day after the injury. Sca-1+ progenitor cells appeared on the first day of injury at the vascular wall media and then gradually increased and reached peak levels on the seventh day after injury. Notably, Sca-1+ progenitor cells in the intima or neointima were not observed at least 3 days after the injury, markedly increased on the seventh day, and peaked 14 days after the injury (Fig. 1f–j and Additional file 1: Figure S1). These results indicated that Sca-1+ progenitor cells showed increased traits in the sequence of adventitia, media, and neointima.

Combining the results of S100B and Sca-1+ progenitor cells, the appearance of Sca-1+ progenitor cells at the adventitia, media, and neointima of the vessel wall were associated with S100B expressions.

### S100B promoted Sca-1+ progenitor cell migration into neointima via RAGE

**Fig. 2:**
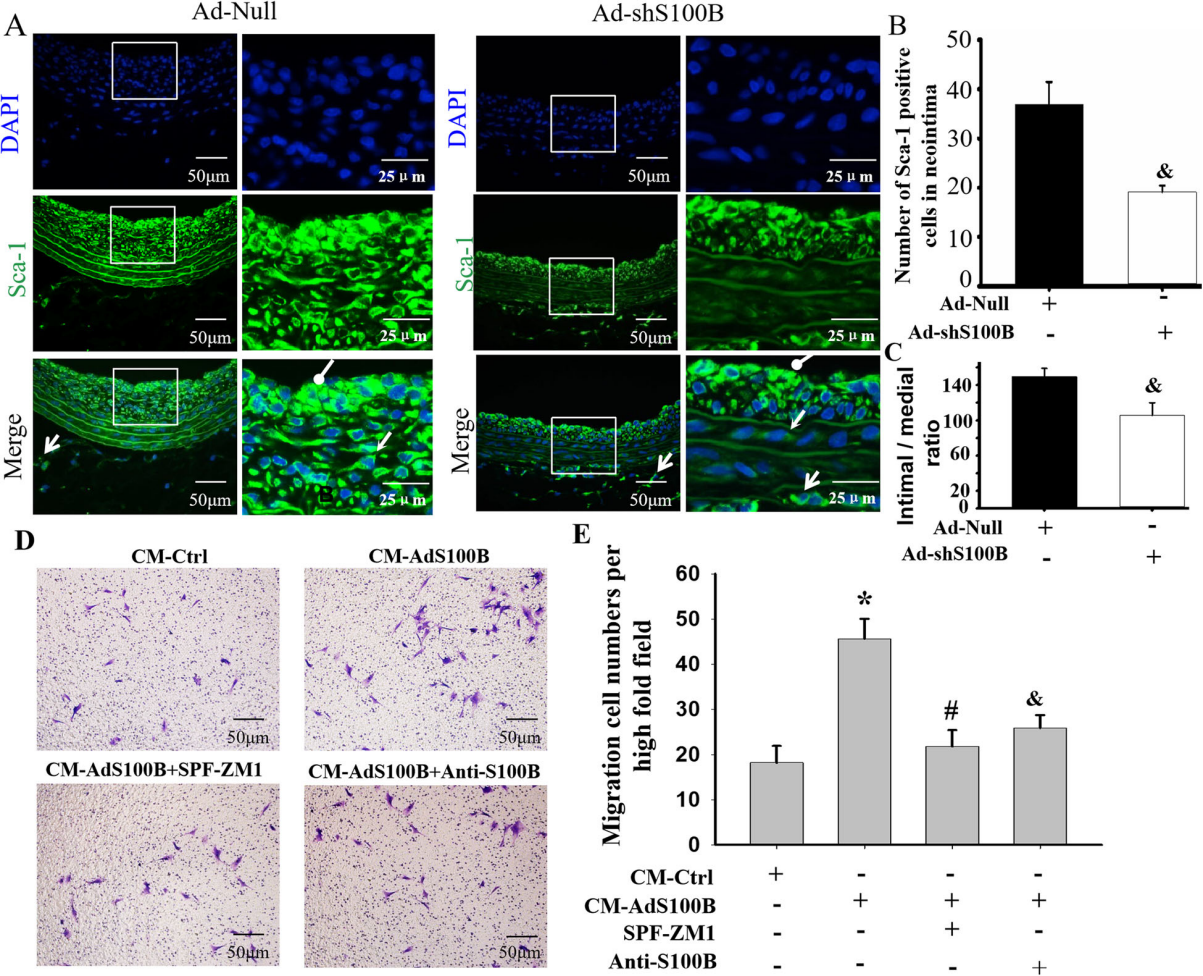
S100B promoted Sca-1+ progenitor cell migration into the neointima through RAGE. Further experiments were designed to corroborate whether the increased S100B in injured vessels induced Sca-1+ progenitor cell migration into the neointima, by shRNA-induced SB100 knockdown in the injured vessels in vivo, using S100B neutralizing antibody or RAGE inhibitor to confirm the effect of S100B on Sca-1+ progenitor cell migration. **a** Representative image of fluorescence staining for Sca-1 in injured arteries treated with or without Ad-shS100B for 7 days. Round arrow indicates the neointima area, open arrow indicates the media, and forked tail arrow indicates the adventitia. Round arrow indicates the neointima area, open arrow indicates the media, and forked tail arrow indicates the adventitia. **b**, **c** The number of Sca-1+ progenitor cells were determined within the media or intima of **a** by image-Pro Plus software. *n* = 6, ^&^*P* < 0.05 vs. the injured arteries treated with Ad-Null. **d** Typical image of response of human umbilical cord Sca-1 progenitor cell migration to conditioned medium (CM) of VSMC treated with or without Ad-S100B, following the addition of neutralizing antibody for S100B or RAGE inhibitor SPF-ZM1. **e** Quantitative analysis of cell migration was analyzed using the Transwell system. *n* = 15, *P < 0.05 vs. CM-Ctrl; ^#^*P* < 0.05 vs. CM-Ad-S100B; ^&^*P* < 0.05 vs. CM-Ad-S100B

To confirm whether S100B was involved in the process of Sca-1+ progenitor cell migration into the neointima, the number of Sca-1+ progenitor cells in the media and neointima were assessed after local application of Ad-shS100B within the balloon-injured carotid artery. As shown in Fig. 2a, b, the numbers of Sca-1+ progenitor cells in Ad-shS100B-treated arteries were prominently decreased not only at the vascular wall media but also at the neointima, as compared with Ad-Null-treated arteries, indicating that S100B participated in the course of Sca-1+ progenitor cell migration into the neointima. Meanwhile, the I/M ratios were lower in Ad-shS100B-treated arteries than Ad-Null-treated arteries (Fig. 2c). These results proved that S100B could trigger neointimal formation by inducing Sca-1+ progenitor cell migration into the neointima.

Given that S100B is a ligand for RAGE, we tested whether released S100B in VSMCs promoted Sca-1+ progenitor cell migration through its receptor RAGE. As seen in Fig. 2d, e and Additional file 1: Figure S1, Sca-1+ progenitor cells expressed RAGE and the migratory response of Sca-1+ progenitor cells to CM of VSMCs treated with Ad-S100B (CM-AdS100B) were substantially increased. The migration action of Sca-1+ progenitor cells mediated by CM-AdS100B was evidently abrogated by FPS-ZM1 (RAGE inhibitor) or neutralizing antibody of S100B, thereby indicating that S100B could induce Sca-1+ progenitor cell migration through RAGE.

Taken together, S100B could trigger neointimal formation by inducing Sca-1+ progenitor cell migration into the neointima through RAGE.

### S100B maintains an intermediate state of double-positive Sca-1+ progenitor cells and VSMCs

**Fig. 3:**
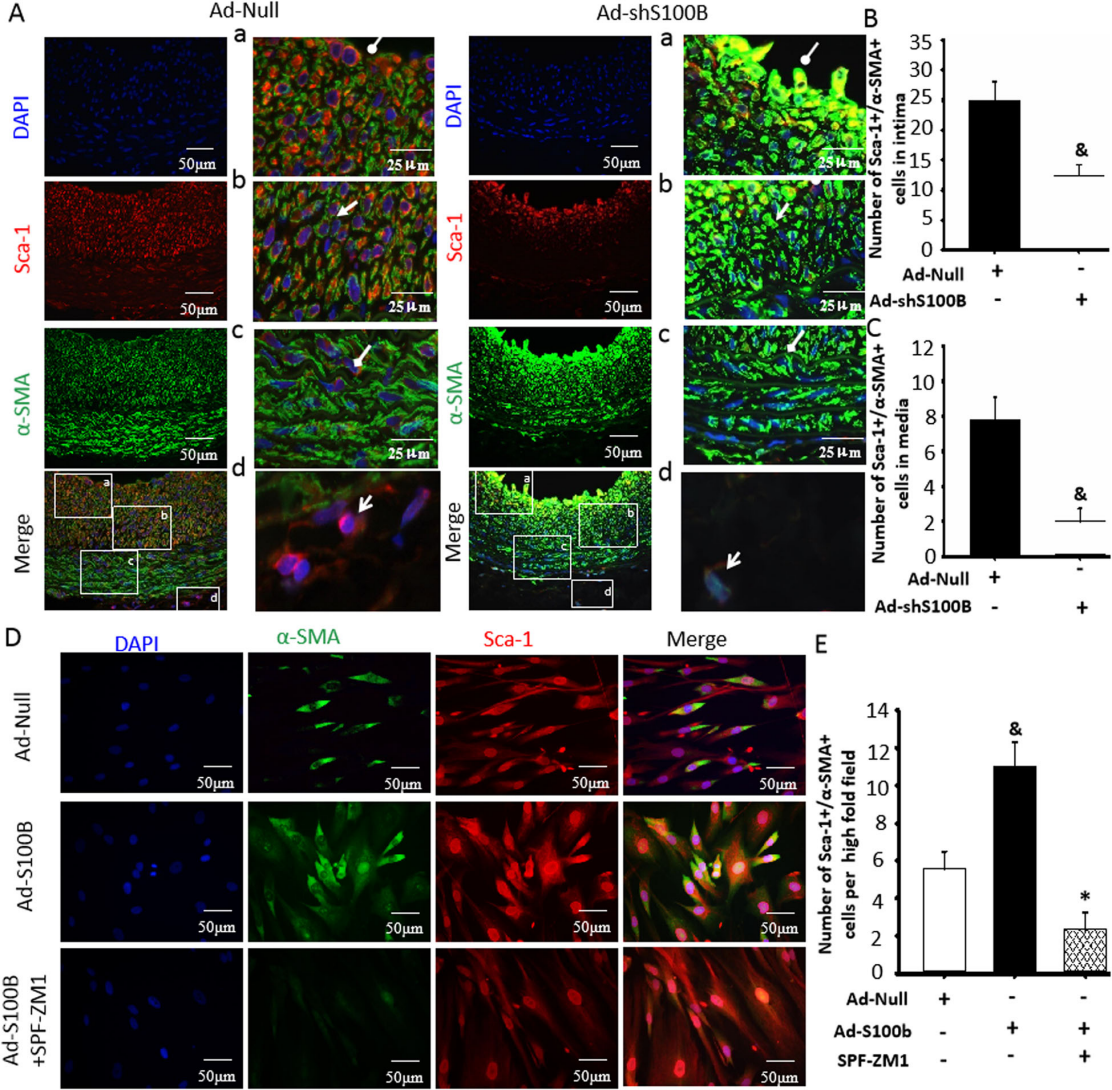
S100B maintains an intermediate state of double-positive Sca-1+ stem cells and VSMCs. This experiment preliminarily demonstrated the existence of an intermediate state of double-positive stem cells and VSMCs in the injured vessels and tried to identify the correlation between the intermediate state and S100B. **a** Representational image of immunofluorescence double staining for Sca-1 and α-SMA in injured arteries treated with or without Ad-shS100B for 14 days. Red fluorescence indicates Sca-1, green fluorescence indicates α-SMA, and blue fluorescence indicates DAPI-labeled nucleus. Round arrow indicates lumen-side neointima, open arrow indicates media-side neointima, diamond arrow indicates the media, and forked tail arrow indicates the adventitia. **b**, **c** The number of double-positive cells with Sca-1 and α-SMA were determined within the media or intima (panel 3A) by image-Pro Plus software. *n* = 6, ^&^*P* < 0.05 vs. the injured arteries treated with Ad-Null. **d** Representative image of immunofluorescence staining for Sca-1 and α-SMA in human umbilical cord-derived Sca-1+ progenitor cells with or without Ad-S100B, following the addition of RAGE inhibitor SPF-ZM1. Red fluorescence indicates Sca-1, green fluorescence indicates α-SMA, and blue fluorescence indicates DAPI-labeled nucleus. **e** Quantitative analysis of immunofluorescence double staining for Sca-1 and α-SMA was analyzed in human umbilical cord Sca-1+ progenitor cells with Ad-S100B, following the addition of RAGE inhibitor SPF-ZM1. *n* = 15, ^&^*P* < 0.05 vs. Ad-Null; *P < 0.05 vs. Ad-S100B

We determined how S100B was involved in neointimal formation, apart from promoting Sca-1+ progenitor cell migration into the vessel neointima. Double immunofluorescence staining of Sca-1 and α-SMA in the artery showed that Sca-1+ progenitor cells did not express α-SMA at the vascular adventitia (Fig. 3a–c). A few Sca-1+ progenitor cells were positive for α-SMA at the tunica media. Of interest, most Sca-1+ progenitor cells in the neointima of injured arteries acquired the phenotype of α-SMA. By contrast, Sca-1+ progenitor cells in the neointima of Ad-shS100B-treated arteries lost the phenotype of Sca-1 while acquiring a stronger phenotype of α-SMA (Fig. 3a–c, Additional file 1: Figure S2). Furthermore, the I/M ratios were obviously reduced in Ad-shS100B-treated arteries (Additional file 1: Figure S3). These results proved that an intermediate state of double-positive Sca-1+ progenitor cells and α-SMA in the injured arteries was essential for neointimal development, which was associated with S100B.

Meanwhile, Sca-1+ progenitor cells from the adventitia of the umbilical cord showed spontaneous differentiation potential into VSMCs with positive α-SMA (Fig. 3D, E), and the effect was obviously enhanced by overexpression of S100B. More importantly, the enhanced differentiation potential by Ad-S100B was clearly canceled by the RAGE inhibitor, SPF-ZM1.

Hence, increased S100B expression is necessary in the development of neointimal formation for maintaining the intermediate state of double-positive Sca-1+ progenitor cells and VSMCs.

### S100B and SDF-1α expression during balloon injury-induced neointimal formation

**Fig. 4:**
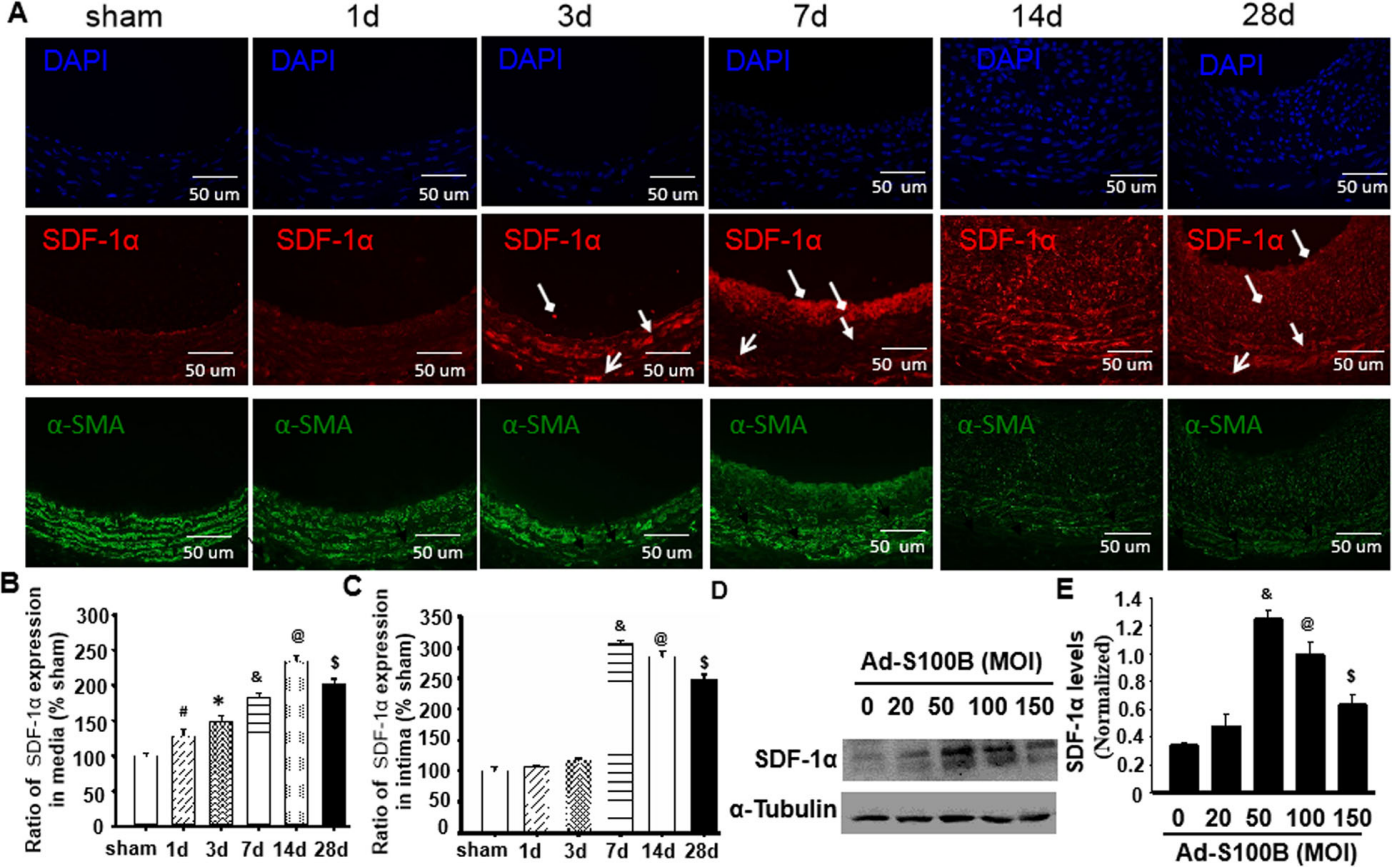
S100B and SDF-1α expression during balloon injury-induced neointimal formation. On the basis of the results of Fig. 1, this experiment preliminarily demonstrated the order of expressions of S100B and SDF-1α in the process of neointimal formation after vessel injury and tried to determine the correlation between expressions of S100B and SDF-1α. **a** Representational image of immunofluorescence double staining for SDF-1α and α-SMA in injured arteries at the indicated time points. Red fluorescence indicates SDF-1α, green fluorescence indicates α-SMA, and blue fluorescence indicates DAPI-labeled nucleus. Round arrow indicates lumen-side neointima, open arrow indicates the media, and forked tail arrow indicates the adventitia. **b**, **c** Semi-quantitative analysis of optical density value of immunohistochemical staining of SDF-1α within the media or intima of sham-operated and injured arteries was carried out using the image-Pro Plus software. The percentages of SDF-1α expression in the media or intima at the indicated time points compared to the sham group were calculated that showed time-dependent characteristics. **d** The dynamics of SDF-1α expression in VSMCs transfected with different MOI Ad-S100B as determined by western blot. **e** Semi-quantitative analysis of SDF-1α in the VSMCs from 4D. *n* = 3, ^&^*P* < 0.05 vs. 30 MOI Ad-S100B; *P < 0.05 vs. 60 MOI Ad-S100B; ^$^*P* < 0.05 vs. 80 MOI Ad-S100B

SDF-1α/CXCR4 plays crucial roles in controlling stem cell migration [29]. We explored whether S100B-induced Sca-1+ progenitor cell migration into the vessel neointima involved SDF-1α/CXCR4. As shown in Fig. 4a–d, following the changed characteristics of S100B in the injured artery, SDF-1α expressions were gradually increased at the vessel wall adventitia in a time-dependent manner and peaked on the third day after injury. SDF-1α expressions were slightly increased on the first day of injury at the vascular wall media and then gradually increased and reached peak levels 14 days after the injury. Of note, SDF-1α expressions in the intima or neointima were not found at least 3 days after injury, markedly increased on the seventh day, and peaked on day 14 after the injury (Fig. 4a–c).

To observe the relationship of S100B and SDF-1α in VSMCs, Ad-S100B was used for transfection into VSMCs with different MOIs. As shown in Fig. 4d, e, S100B induced the expression of SDF-1α in VSMCs in an MOI-dependent manner, reaching peak values at 50 MOI.

Combining the results of S100B and Sca-1+ progenitor cells (Figs. 1 and 2), SDF-1α could participate in the process of S100B-mediated Sca-1+ progenitor cell migration into the neointima upon arterial injury.

### S100B knockdown reduced Sca-1+ progenitor cell migration into the neointima through SDF-1α/CXCR4

**Fig. 5:**
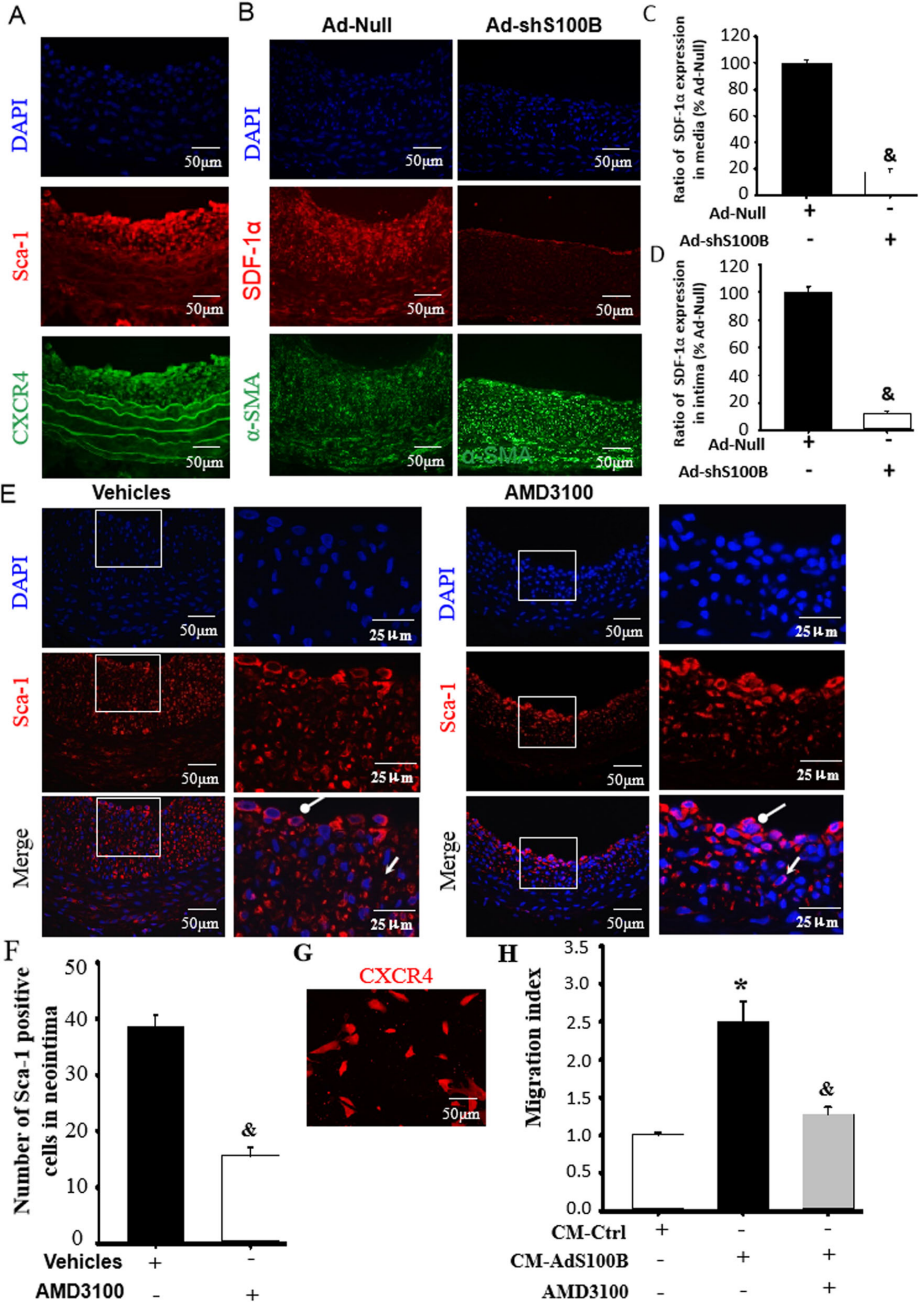
S100B reduced Sca-1+ progenitor cell migration into the neointima through SDF-1α/CXCR4. The experiments were designed to determine whether increased SDF-1α in injured vessels induced Sca-1+ progenitor cell migration into the neointima by using SDF-1α receptor CXCR4 blocker AMD3100 to affirm the role of SDF-1α on Sca-1+ progenitor cell migration 7 days after vessel injury with or without AMD3100 application in vivo and 12 h after treatment in vitro. **a** Representational image of immunofluorescence double staining for Sca-1 and CXCR4 in injured arteries for 7 days. Red fluorescence indicates Sca-1, green fluorescence indicates CXCR4, and blue fluorescence indicates DAPI-labeled nucleus. **b**–**d** Typical image and semi-quantitative analysis of double positive with SDF-1α and α-SMA were determined within the media or intima of injured arteries treated with Ad-shS100B for 14 days, using the image-Pro Plus software. Red fluorescence indicates SDF-1α, green fluorescence indicates α-SMA, and blue fluorescence indicates DAPI-labeled nucleus. *n* = 6, ^&^*P* < 0.05 vs. the injured arteries treated with Ad-Null. **e** Representative image of immunofluorescence staining of Sca-1 in the neointima of injured arteries treated with AMD3100. Red fluorescence indicates Sca-1, and blue fluorescence indicates DAPI-labeled nucleus. Round arrow indicates the neointima area, and open arrow indicates the media. **f** AMD3100 decreased the number of Sca-1+ progenitor cells in the neointima (panel **e**). *n* = 6, ^&^*P* < 0.05 vs. Ad-Null. **g** Typical image of CXCR4 expression in hUCMSCs. Red fluorescence indicates CXCR4, and blue fluorescence indicates DAPI-labeled nucleus. **h** Cell migration mediated by conditioned medium (CM) with Ad-S100B was analyzed using the Transwell system following additions with or without of AMD3100. *n* = 15, *P < 0.05 vs. CM-Ctrl; ^&^*P* < 0.05 vs. CM-Ad-S100B

SDF-1α can trigger cell migration through CXCR4 [26]. To confirm whether S100B was involved in CXCR4 in Sca-1+ progenitor cells, we used immunofluorescence staining (Fig. 5a) and found that Sca-1+ progenitor cells in the vascular adventitia expressed CXCR4. Considering the evidence of S100B-induced SDF-1α in VSMCs (Fig. 2), we found that knockdown of S100B by shRNA in the injured arteries reduced SDF-1α levels in the VSMCs of the vessel wall neointima and media (Fig. 5b–d). More importantly, when the injured arteries were treated with AMD3100, the numbers of Sca-1+ progenitor cells were dramatically reduced in the vascular wall media and neointima (Fig. 5e, f). Finally, to further corroborate whether CXCR4 in Sca-1+ progenitor cells referred to released SDF-1α-mediated cell migration by S100B, Sca-1+ progenitor cells from human umbilical cord adventitia were cultured and showed traits of CXCR4 expression (Fig. 5g). Furthermore, the migratory response of Sca-1 progenitor cells to CM of VSMCs treated with Ad-S100B (CM-AdS100B) was evidently abrogated by CXCR4 blocker, AMD3100, thereby indicating that released SDF-1α by S100B induced Sca-1+ progenitor cell migration through CXCR4 (Fig. 5h).

Thus, the process of S100B-mediated Sca-1+ progenitor cell migration into the neointima of injured arteries involved SDF-1α/CXCR4.

### S100B maintains the intermediate state of double-positive Sca-1+ progenitor cells and VSMCs through SDF-1α/CXCR4

**Fig. 6:**
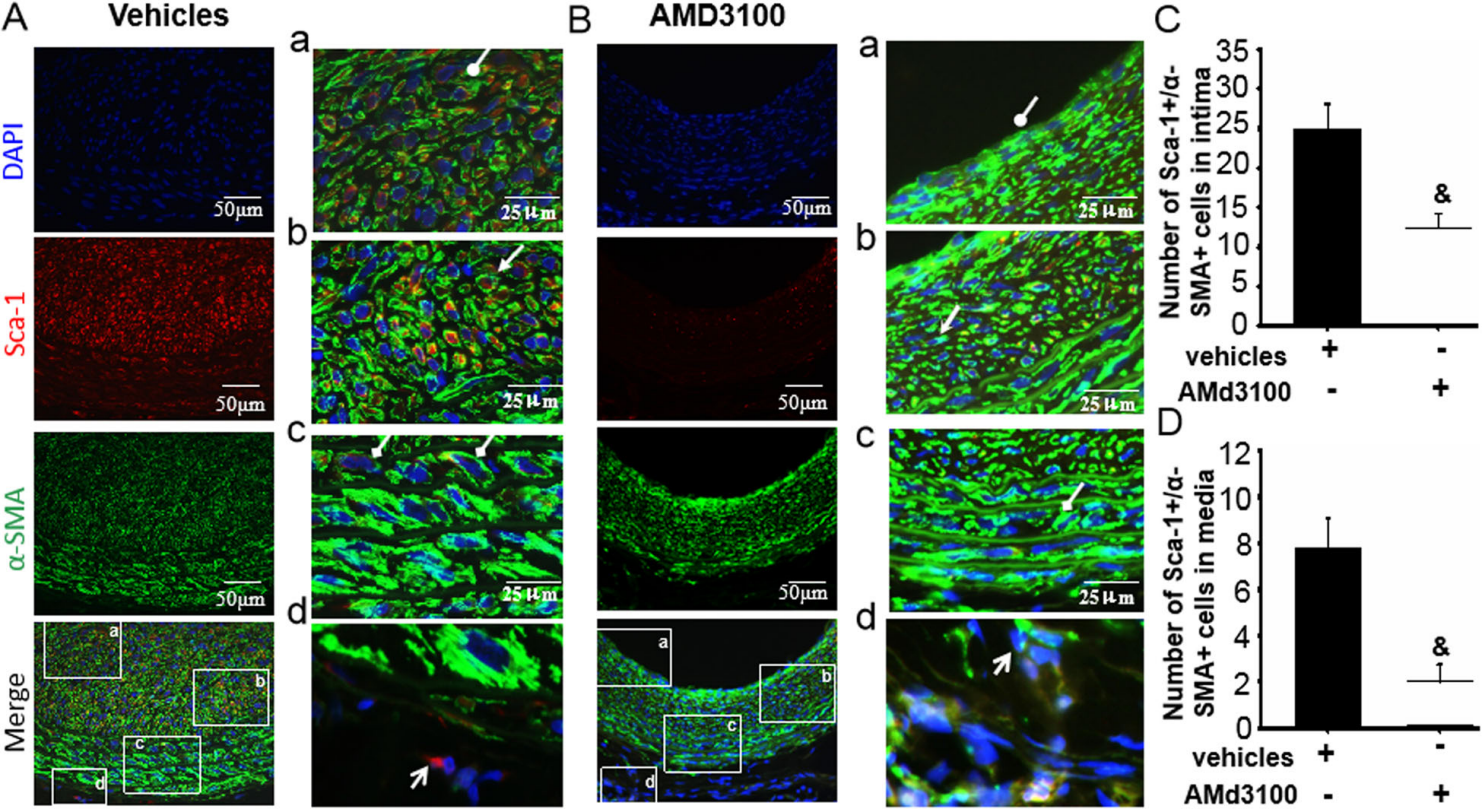
S100B maintains an intermediate state of double-positive Sca-1+ stem cells and VSMCs through SDF-1α/CXCR4. This experiment was designed to demonstrate the role of SDF-1α/CXCR4 axis in maintaining an intermediate state of stem cells and VSMCs in the injured vessels and attempted to show indirect confirmation of the relationship between the role of S100B and intermediate state, likely involving SDF-1α/CXCR4. **a**, **b** Typical image of immunofluorescence double staining for Sca-1 and α-SMA in injured arteries treated with or without AMD3100 for 14 days. Red fluorescence indicates Sca-1, green fluorescence indicates α-SMA, and blue fluorescence indicates DAPI-labeled nucleus. Round arrow indicates lumen-side neointima, open arrow indicates media-side neointima, diamond arrow indicates the media, and forked tail arrow indicates the adventitia. **c**, **a** The number of double-positive cells with Sca-1 and α-SMA within the media or intima (seen in **a**, **b**) was determined by image-Pro Plus software. *n* = 6, ^&^*P* < 0.05 vs. the injured arteries treated with Ad-Null

Considering the special role of S100B in maintaining the intermediate state of double-positive Sca-1+ progenitor cells and VSMCs, it was necessary to assess the involvement of S100B-mediated SDF-1α/CXCR4 activation. Double immunofluorescence staining of Sca-1+ and α-SMA in the artery showed that Sca-1+ progenitor cells showed very faint expression of α-SMA at the vascular adventitia of injured arteries (Fig. 6a), but almost acquired phenotype of α-SMA in the AMD3100 group (Fig. 6b). Interestingly, a few Sca-1+ progenitor cells in the vehicle group were present and showed α-SMA expression at the tunica media. However, there were very few Sca-1+ progenitor cells in the AMD3100 group that simultaneously expressed α-SMA. More importantly, most Sca-1+ progenitor cells in the neointima of the vehicle groups acquired the α-SMA phenotype. By contrast, cells in the neointima of AMD3100-treated arteries lost the Sca-1+ phenotype and acquired stronger α-SMA phenotype because of less Sca-1+ progenitor cells and more α-SMA+ cells (Fig. 6a–d, Additional file 1: Figure S4). Furthermore, the I/M ratios in AMD3100-treated arteries were obviously reduced (Additional file 1: Figure S5). These results showed that the maintained intermediate state of double-positive Sca-1+ progenitor cells and VSMCs by S100B at least partly involved SDF-1α/CXCR4.

### S100B induced SDF-1α expression in VSMCs through NF-kB signaling

**Fig. 7:**
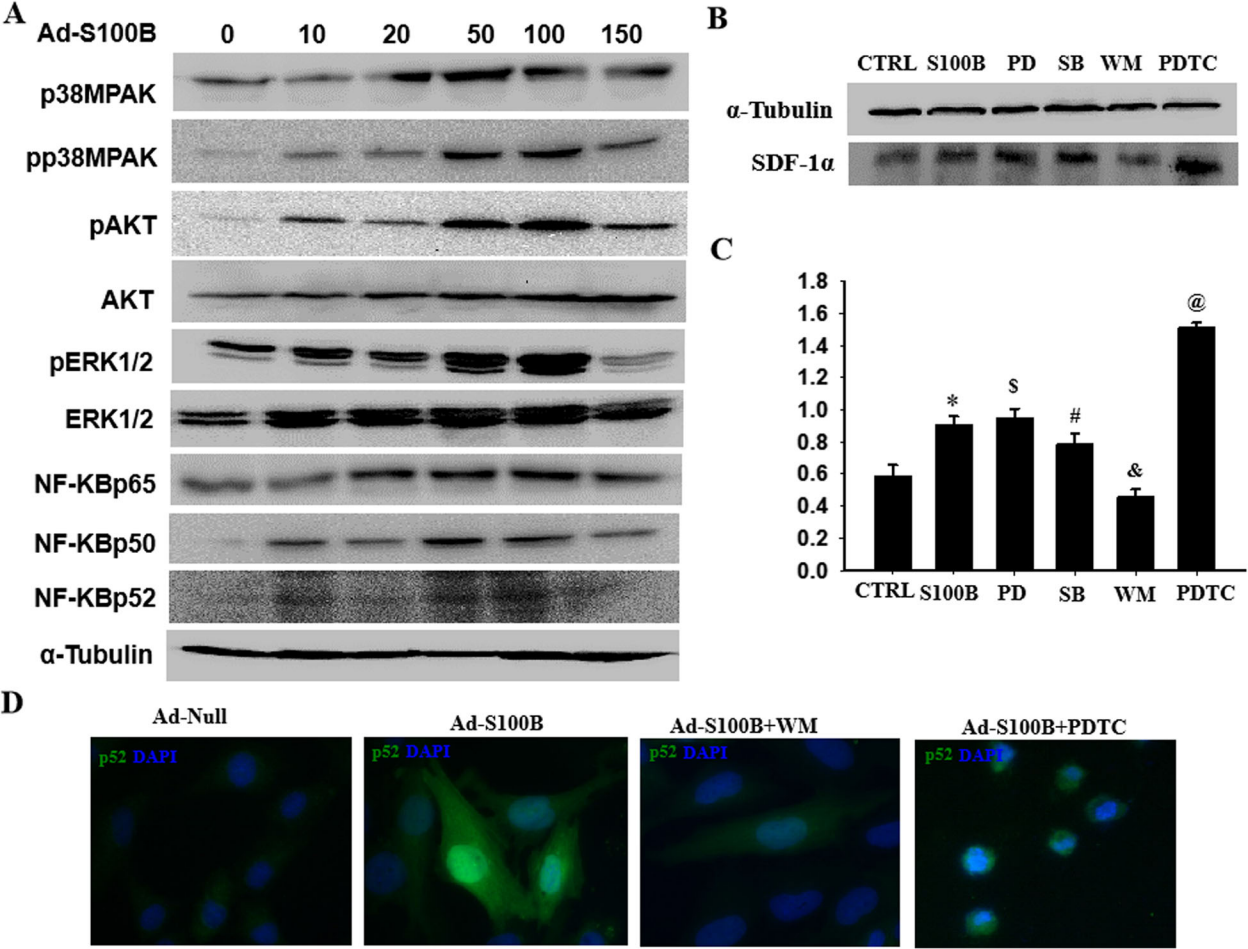
S100B induced SDF-1α expression in VSMCs through NF-kB signaling. The aim of the experiment was to explore possible molecular mechanisms of S100B-induced SDF-1α expression in VSMCs, using blockers for multiple signal pathways. **a** The indicated proteins and phosphorylated proteins as determined by western blot. **b**, **c** The SDF-1α expressions in VSMCs transfected with Ad-S100B, following the addition of ERK1/2 inhibitor PD98059 (PD), p38MAPK inhibitor SB203580 (SB), PI3K/Akt inhibitor wortmannin (WM), or NF-kB blocker (PDTC), as determined by western blot. *n* = 3, *P < 0.05 vs. Ad-Null; ^$^*P >* 0.05 vs. Ad-Null; ^#^*P* < 0.05 vs. Ad-S100B; ^&^*P* < 0.001vs. Ad-S100B; ^@^*P* < 0.001 vs. Ad-S100B. **d** Typical image of immunofluorescence staining for p52 in S100B-overexpressing VSMCs treated with or without PDTC or WM for 3 days. Green fluorescence indicates p52, and blue fluorescence indicates DAPI-labeled nucleus

To further determine how S100B regulated SDF-1α expression in VSMCs, multiple signaling pathways were detected in S100B-overexpressingVSMCs. As shown in Fig. 7a and Additional file 1: Figure S6, overexpression of S100B did not change the total levels of ERK1/2 and Akt, but increased pAkt, pERK1/2, and pp38MAPK levels. Meanwhile, S100B-induced SDF-1α expression in VSMCs could not be abolished by ERK1/2 inhibitor PD98059 or p38MAPK inhibitor SB203580 but was eliminated by PI3K/Akt inhibitor wortmannin. Notably, SDF-1α expression mediated by S100B in VSMCs could be enhanced by NF-κB blocker PDTC (Fig. 7b, c). Indeed, S100B activated NF-κB p65 and p52 by promoting nuclear translocation of p65 and p52. As shown in Fig. 7d, overexpression of S100B induced p52 nuclear translocation; these specific effects could obviously be abolished by wortmannin and enhanced by PDTC. These results proved that NF-kB and PI3K/Akt signal pathways referred to S100B-induced SDF-1α expression in VSMCs.

## Discussion

To our best knowledge, this study presented three novel findings. First, S100B triggered adventitia Sca-1+ progenitor cell migration into neointimal and accelerated neointimal formation through binding into RAGE. Secondly, SDF-1α induced by S100B in VSMC promoted Sca-1+ progenitor cell migration through activating CXCR4. And lastly, for the first time, S100B maintained the intermediate state of double positive of Sca-1+ progenitor cells and VSMCs through activating RAGE and/or SDF-1α/CXCR4, which were associated with PI3K/Akt and NF-kB signaling pathways.

Published data have shown that the mobilization and recruitment of abundant Sca-1+ progenitor cells from the adventitia and media of vascular walls were mainly responsible for intimal VSMC accumulation during vascular remodeling such as intimal hyperplasia and arterial sclerosis [5, 6, 8, 30–34]. Herein, our study showed that Sca-1+ progenitor cells not only existed in the adventitia and media but also migrated to the intima during vascular injury and participate in the formation of neointima. Furthermore, we reported for the first time to our best knowledge that Sca-1+ progenitor cells existed within the adventitia and media on the first day after vessel injury; there were more Sca-1+ progenitor cells at the adventitia than at the media on the third day of vessel injury, and peaked at the neointima on the seventh day of vessel injury, indicating that adventitial Sca-1+ progenitor cells in damaged vessels migrate in a time-dependent manner into the intima and were involved in the process of neointimal formation.

The roles of S100B/RAGE in stem cell proliferation and differentiation have been previously observed [16–19, 35]. Our previous study showed that S100B/RAGE participated in neointimal formation as a result of induced VSMC accumulation [13]. The present study provided new evidence that S100B expression was greatest at the adventitia on the first day after vessel injury; S100B levels gradually increased from the media to the neointima in a time-dependent manner after vascular injury. Moreover, along with the increasing trend of S100B expression in the sequence of the three vascular layers, the numbers of Sca-1+ progenitor cells were prominently increased at the adventitia, media, or neointima during the time course of neointimal formation. By contrast, knockdown of S100B by shRNA obviously decreased the numbers of Sca-1+ progenitor cells within the neointima in vivo, and the RAGE blocker markedly eliminated its migration induced by CM treated with Ad-S100B in vitro. These results suggested that S100B could directly trigger Sca-1+ progenitor cell migration and neointimal formation by activating RAGE.

In addition to the gradually increased trends of S100B, our results have shown that SDF-1α expressions increased gradually in the injured vessels, and SDF-1α was later S100B at the adventitia, consistent with previous studies that serum S100B reached its peak before SDF-lα in case of cardiac and neuronal injuries [21–24]. Subsequently, SDF-1α expressions were not only increased within the media or neointima, accompanied by in vivo increase of S100B, but the VSMCs also showed a gradual increase of SDF-lα levels with increased MOI Ad-S100B in vitro. More importantly, local application of Ad-shS100B in the injured vessels significantly reduced SDF-lα expression in the neointima. Furthermore, NF-κB p65 inhibitor PDTC enhanced the expression of S100B-induced SDF-1α in VSMCs, which was different from the role of activated NF-κB p65 by RAGE in inducing SDF-1α expression in the diabetic kidney [36]. NF-κBp52 activation requires Akt-mediated phosphorylation and nuclear translocation [37–40], which was consistent with our results in that blockade of Akt using wortmannin could completely abolish S100B-induced SDF-1α expression. These results indicated that S100B was a likely inducer of SDF-lα, which could be related to Akt-NF-κBp65-p52 signaling.

The SDF-1α/CXCR4 axis played a crucial role in neointimal formation through activating Sca-1+ progenitor cells [41, 42]. Our results further showed that the knockdown of S100B by shRNA not only reduced the levels of SDF-lα in injured vessels but also decreased the numbers of Sca-1+ progenitor cells at the neointima, leading to inhibition of its formation. Furthermore, AMD3100, as a CXCR4 inhibitor, also reduced cell numbers and neointimal area in vivo and partially abrogated its migration induced by CM treated with Ad-S100B in vitro. In short, these results demonstrated that S100B could indirectly induce Sca-1+ progenitor cell migration and neointimal formation by manipulating the SDF-1α/CXCR4 axis.

Interestingly, VSMCs in the vessel wall were mostly quiescent but could exhibit a contractile phenotype in adults. Under pathophysiological conditions such as vascular injury, contractile VSMCs presented in the media switched to a proliferative phenotype that could facilitate their ability to migrate to the intima and proliferate to contribute to neointimal formation. Considering the heterogeneity of VSMCs [2, 43], a recent unique phenomenon was noted wherein differentiated VSMCs in the outer media could migrate into the inner adventitia and acquire Sca-1 expression, as one of the progenitor cell markers [10, 11], while simultaneously losing the VSMC phenotype; this provided novel evidence for involvement of a small VSMC subpopulation in neointimal formation, apart from the VSMC phenotype switch. However, the origin and differentiation of VSMCs from Sca-1+ progenitor cells during cardiovascular development and in adults have been identified in the vascular wall adventitia and media [44]. Regardless of the differences in the abovementioned views, unique features of the intermediate state with double-positive Sca-1+ progenitor cells and VSMCs could play an important role in maintaining and promoting neointimal formation after vessel injury. Indeed, previous studies have shown that acquiring and maintaining the intermediate state were required during stem cell differentiation [22–24]. The present study not only identified the intermediate state of Sca-1 and α-SMA in the injured artery but also the involvement of S100B in acquiring and maintaining the intermediate state. In line with the role of S100B in regulating stemness of ovarian cancer stem-like cells [45, 46], when knocking down S100B, Sca-1+ progenitor cells within the media and neointimal lost the progenitor cell traits and acquired more contractile phenotypes of VSMCs including α-SMA, leading to the reduction of neointimal area and thickness. Furthermore, S100B-induced SDF-1α referred to preservation of the intermediate state, and the specific effects could be in part abrogated by AMD3100, which was similar to the role of AMD3100 in promoting differentiation of stem cells into cardiomyocytes [47–49].

## Conclusions

**Fig. 8:**
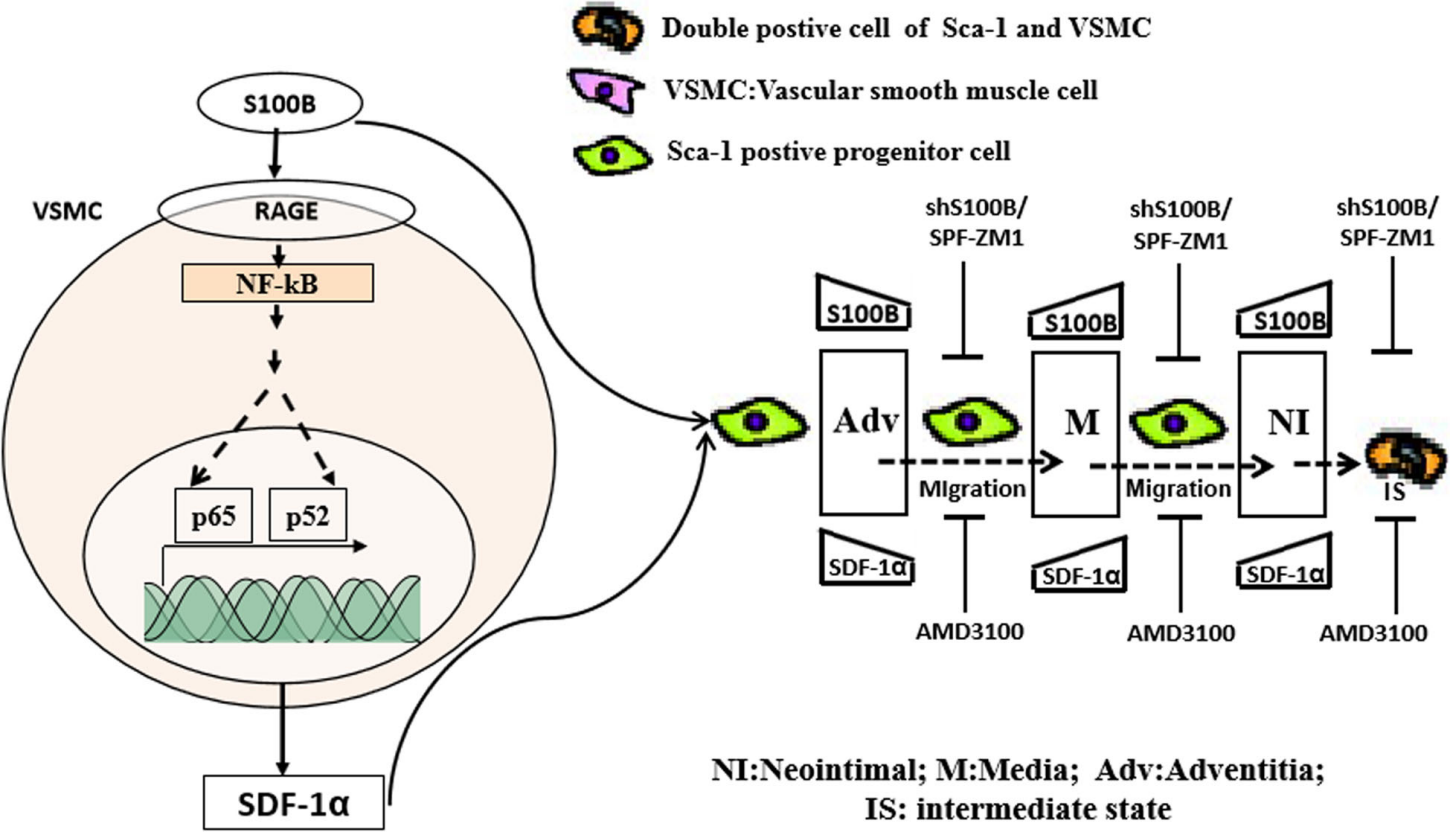
Working model of S100B the process of neointimal formation. S100B triggered neointimal formation in the injured arteries by maintaining the intermediate state of double-positive Sca-1+ progenitor cells and VSMCs that was associated with RAGE and/or SDF-1α/CXCR4

Increased Sca-1+ progenitor cells gradually appeared in the sequence of adventitia, media, and neointima after vessel injury, accompanied by induced S100B expressions in the corresponding vessel wall locations during the process of neointimal formation. Furthermore, S100B triggered neointimal formation in the injured arteries by maintaining an intermediate state of double-positive stem cells and VSMCs that was associated with RAGE and/or SDF-1α/CXCR4 (Fig. 8).

## Additional file

Additional file 1: **Figure S1.** Sca-1+ progenitor cells were increased in the sequence of adventitia, media, and neointima, showing the traits of RAGE expression during balloon injury-induced neointimal formation. **Figure S2.** S100B knockdown by shRNA reduced the number of Sca-1+/α-SMA- cells while increasing Sca-1−/α-SMA+ cells within the media or intima determined by image-Pro Plus software. **Figure S3.** S100B knockdown on by shRNA decreased the I/M ratios of the injured arteries. **Figure S4.** AMD3100 reduced the number of Sca-1+/α-SMA- cells while increasing Sca-1−/α-SMA+ cells within the media or intima. **Figure S5.** AMD3100 decreased the I/M ratios of the injured arteries. Figure S6. Semi-quantitative assay for the indicated proteins and phosphorylated proteins as determined by western blot. (DOCX 1427 kb)

## Data Availability

Please contact the corresponding author for data requests.

## Abbreviations

Adv: Adventitial
AKT: Protein kinase B
CAD: Coronary artery disease
CXCR4: C-X-C motif chemokine receptor 4
DAPI: 4′,6-Diamidino-2-phenylindole
ERK1/2: Extracellular signal-regulated kinase1/2
HRP: Horseradish peroxidase
hUCMSCs: Human umbilical cord mesenchymal stem cells
IF: Immunofluorescence
M: Mol/L
MAPK: Mitogen-activated protein kinase
MOI: Multiplication of infection
NF-κB: Nuclear Factor Kappa-B
PBS: Phosphate-buffered saline
PCI: Percutaneous coronary intervention
PVDF: Polyvinylidene fluoride
RAGE: Receptor for advanced glycation end products
S100B: S100 calcium-binding protein B
Sca-1: Stem cell antigen-1
SDF-1α: Stromal cell-derived factor 1 alpha
VSMC: Vascular smooth muscle cell
α-SMA: Alpha smooth muscle actin
