# Supplementary material for: S100B is required for maintaining an intermediate state with double-positive Sca-1+ progenitor and vascular smooth muscle cells during neointimal formation
Source: Stem Cell Res Ther. 2019 Sep 23;10:294. doi: 10.1186/s13287-019-1400-0 (PMC6757428; doi:10.1186/s13287-019-1400-0)
Supplement: Supplementary file 1 — Figure S1. Sca-1+ progenitor cells were increased in the sequence of adventitia, media, and neointima, showing the traits of RAGE expression during balloon injury-induced neointimal formation. Figure S2. S100B knockdown by shRNA reduced the number of Sca-1+/α-SMA- cells while increasing Sca-1−/α-SMA+ cells within the media or intima determined by image-Pro Plus software. Figure S3. S100B knockdown on by shRNA decreased the I/M ratios of the injured arteries. Figure S4. AMD3100 reduced the number of Sca-1+/α-SMA- cells while increasing Sca-1−/α-SMA+ cells within the media or intima. Figure S5. AMD3100 decreased the I/M ratios of the injured arteries. Figure S6. Semi-quantitative assay for the indicated proteins and phosphorylated proteins as determined by western blot. (DOCX 1427 kb) [file 13287_2019_1400_MOESM1_ESM.docx]

**S100B is required for maintaining an intermediate state with double positive Sca-1+ progenitor and vascular smooth muscle cells during neointimal formation**

Yan Wu^1,4^, Xin Liu^3^, Ling-Yun Guo^2^, Lei Zhang^2,4^, Fei Zheng^2^, Shan Li^5^, Xing-Yuan Li^1^, Ye Yuan^2,4^, Yu Liu^1^, Yu-wen Yan^1^, Shi-You Chen^6^, Jia-Ning Wang^1,4^, Jin-xuan Zhang^1, 4#^, Jun-Ming Tang^1,2,4#^

^1^Department of Physiology, School of Basic Medicine Science, Hubei University of Medicine, Hubei, 442000, China

^2^Institute of Clinical Medicine and Department of Cardiology, Renmin Hospital, Hubei University of Medicine, Shiyan, Hubei 442000, China

^3^Laboratory Animal Center, Hubei University of Medicine, Hubei442000, China

^4^Institute of Biomedicine and Key Lab of Human Embryonic Stem Cell of Hubei Province, Hubei University of Medicine, Hubei 442000, China

^5^Department of Biochemistry, School of Basic Medicine Science, Hubei University of Medicine, Hubei, 442000, China

^6^Department of Physiology & Pharmacology, The University of Georgia, Athens, GA 30602, USA

Yan Wu, E-mail: 2668224536@qq.com

^#^Corresponding author: Jun-Ming Tang, MD, PhD

Tel.: +86-719/8637706; Fax: 86-719/8637792;

1. mail: [tangjm416@163.com](mailto:tangjm416@163.com)

**Supplmental Method**

**Immunofluorescent staining**

To confirm the dynamic process of Sca-1+ progenitor cell migration from the adventitia to intima during the process of neointima formation, the artery sections from different time points were incubated with Sca-1 (ab4336, Abcam), RAGE (ab54741, Abcam) antibody followed by fluorescent dye-conjugated secondary antibody (Jackson ImmunoResearch) and counterstained with DAPI (Sigma).

To observe the expression of RAGE in Sca-1+ progenitor cells after the vessels were injured *in vivo*, the sections were rinsed with PBS and fixed with 4% paraformaldehyde, then blocked with 5% goat serum, permeabilized with 0.01% Triton X-100 in PBS, and incubated with anti-Sca-1 (ab4336, Abcam) and anti-RAGE (ab54741, Abcam) followed followed by fluorescent dye-conjugated secondary antibody (Jackson ImmunoResearch) and counterstained with DAPI (Sigma).

To observe the expression of RAGE in Sca-1+ progenitor cells *in vitro*, Sca-1+ stem cells cultured on coverslips were rinsed with PBS and fixed with 4% paraformaldehyde, then blocked with 5% goat serum, permeabilized with 0.01% Triton X-100 in PBS, and incubated with anti-Sca-1 and anti-RAGE followed by fluorescent dye-conjugated secondary antibody (Jackson ImmunoResearch) and counterstained with DAPI (Sigma).

**Flow cytometry analysis**

200,000 to 300,000 third passge hUCMSCs were plated into each well of a 96-well plate. The cells were then resuspended in 30 μL of primary antibodies for one hour at 4°C. The primary antibodies and dilutions are described above for ICC with the addition of FITC-CD34 (323203,biolegeng), FITC-CD105(328107,biolegeng), FITC-CD90(344015,biolegeng),FITC-CD73(304005,biolegeng), FITC-CD45(101205, biolegeng), FITC-CD11b(400107,biolegeng) or FITC-mouse IgG1 Isotype ctrl(343503,biolegeng). The cells were again rinsed twice and fixed using 4% paraformaldehyde for ten minutes on ice. The cells were rinsed and stored at 4°C until analysis was performed (BD Bioscience).

**Supplemental Figure 1**


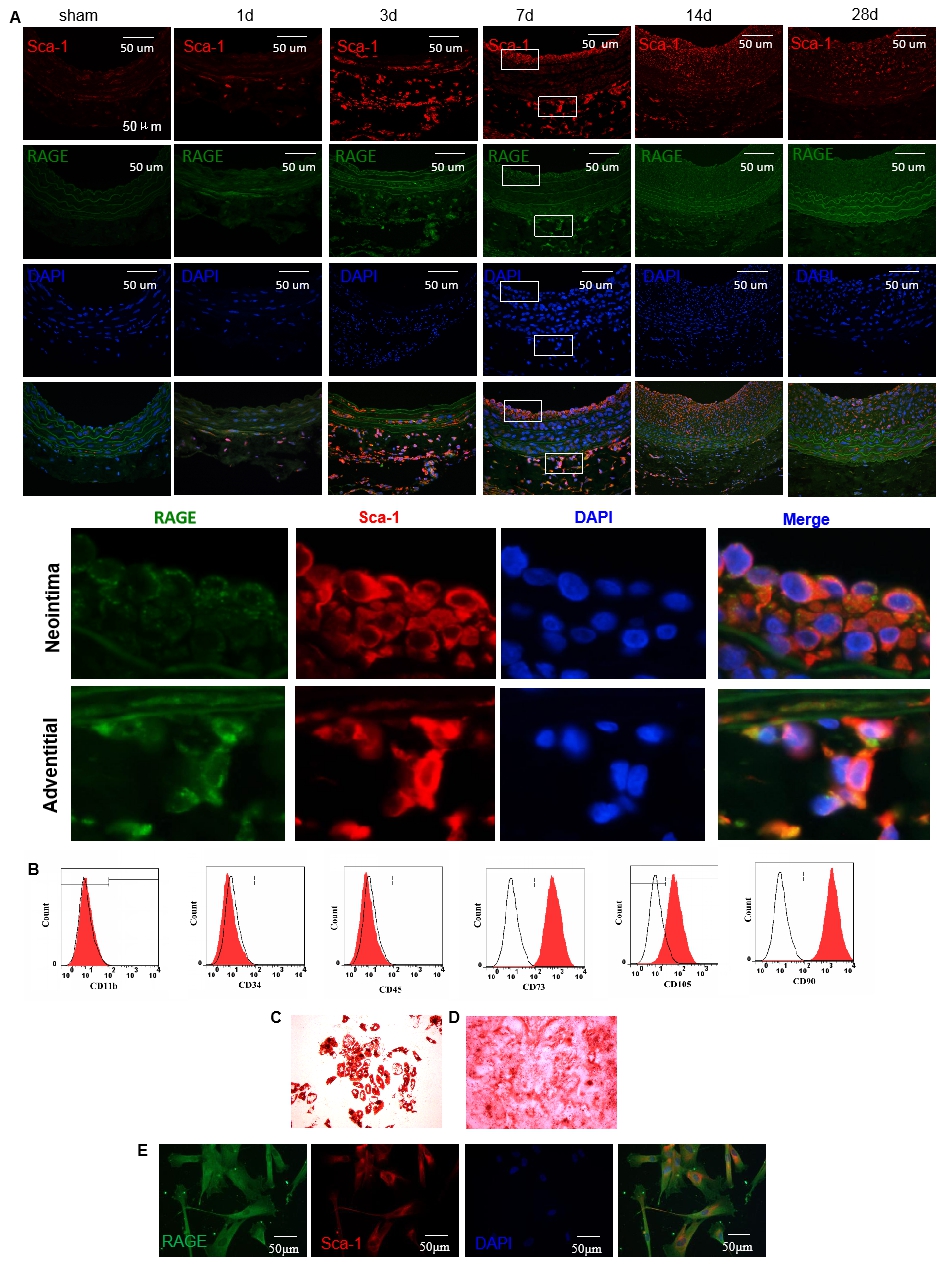


**The traits of Sca-1+progenitor cells and RAGE expression during balloon injury-induced neointimal formation**

(A)Typical image of immunofluorescence double staining with Sca-1 and RAGE in sham-operated and injured arteries at the indicated time points. Red fluorescence indicates Sca-1, green fluorescence indicates RAGE, and blue fluorescence indicates DAPI-labeled nucleus.(B)Surface marker assay by FACS showed that hUCMSCs expressed CD1b (0.18%), CD34(0.15%), CD45 (0.16%), CD73( 99.4%), CD90 (99.9%) and CD105(89.8%).(C)Typical image of immunofluorescence double staining with Sca-1 and RAGE in hUCMSCs. Red fluorescence indicates Sca-1, green fluorescence indicates RAGE, and blue fluorescence indicates DAPI-labeled nucleus.

Quantitative analysis showed that 40% of human umbilical cord mesenchymal stem cells (MSCs) were postive for Sca-1. (C) Typical image of adipogenic differentiation of hUCMSCs. (D) typical image of osteogenic differentiation of hUCMSCs. (E) Typical image of immunofluorescence double staining with Sca-1 and RAGE in hUCMSCs. Red fluorescence indicates Sca-1, green fluorescence indicates RAGE, and blue fluorescence indicates DAPI-labeled nucleus.

**Supplemental Figure 2**


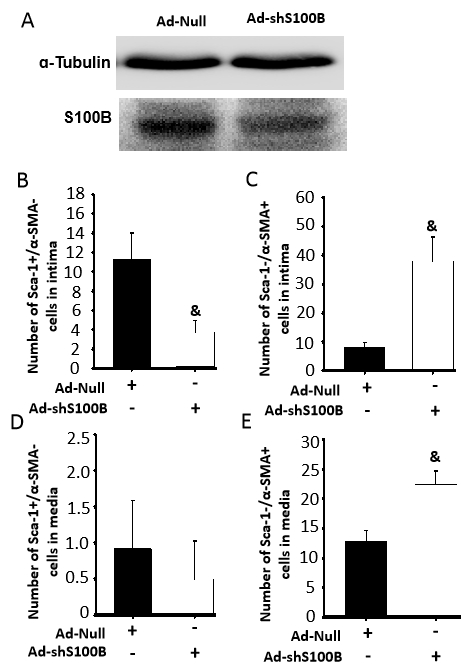


1. Local appication of Ad-shS100B obviously reduced levels of S100B in injured vessel at fourteenth day. (B-E)The number of Sca-1+/α-SMA- or Sca-1-/α-SMA+ cells were determined within the media or intima by image-Pro Plus software. n=6, ^&^*P*<0.05 vs. the injured arteries treated with Ad-Null.

**Supplemental Figure 3**


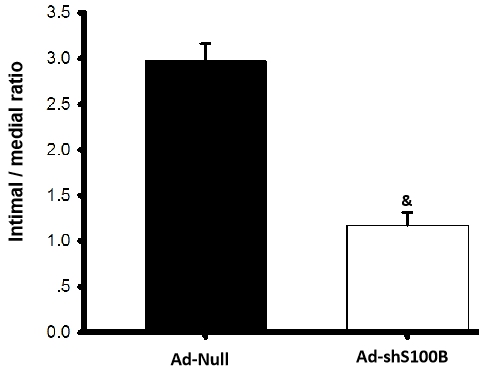


The effect of S100B knockdownon on the I/M ratios of the injuried arteries. n=6, ^&^*P*<0.05 compared to Ad-Null group.

**Supplemental Figure 4**


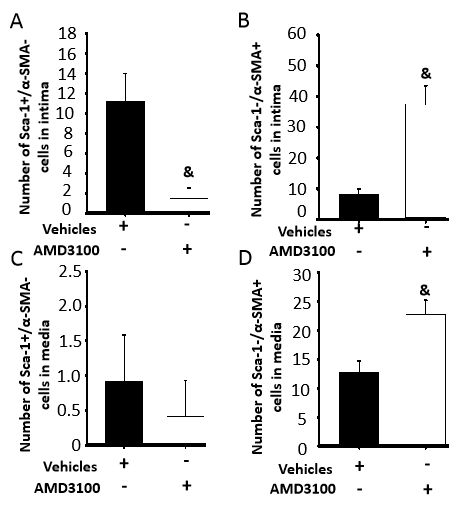


The number of Sca-1+/α-SMA- or Sca-1-/α-SMA+ cells were determined within the media or intima by image-Pro Plus software. n=6, ^&^*P*<0.05 vs. the injured arteries treated with vehicle.

**Supplemental Figure 5**


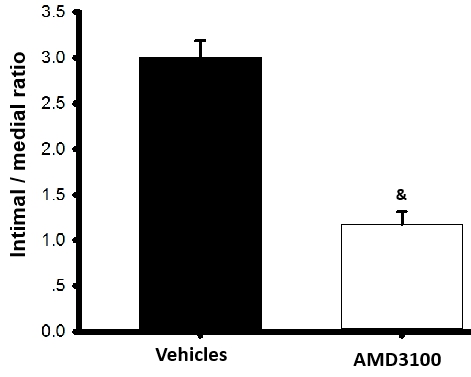


The effect of AMD3100 on the I/M ratios of the injuried arteries. n=6, ^&^*P*<0.05 compared to Vehicles group.

**Supplemental Figure 6**

Semi-quantitative assay for the indicated proteins and phosphorylated proteins as determined by western blot. n=3, ^#^*P*<0.05 compared to 0 MOI; ^*^*P*<0.05 vs. 0 MOI or 10 MOI; ^&^*P*<0.05 vs. 0 MOI or 20 MOI; ^@^*P*<0.05 vs. 0 MOI or 50 MOI; and ^$^*P*<0.05 vs 100 MOI.
